# Editorial: Pharmacological and nutritional approaches to metabolic associated fatty liver disease: a step towards achieving SDG 3

**DOI:** 10.3389/fphar.2026.1802444

**Published:** 2026-02-17

**Authors:** Mahmut Bodur, Bojana Vidović, Anastasios Nikolaou, Birsen Yilmaz

**Affiliations:** 1 Department of Nutrition and Dietetics, Faculty of Health Sciences, Ankara University, Ankara, Türkiye; 2 Department of Bromatology, Faculty of Pharmacy, University of Belgrade, Belgrade, Serbia; 3 Department of Molecular Biology and Genetics, Democritus University of Thrace, Alexandroupolis, Greece; 4 Department of Biological Sciences, Tata Institute of Fundamental Research, Hyderabad, India; 5 Advanced Research Unit on Metabolism, Development and Ageing (ARUMDA), Tata Institute of Fundamental Research, Hyderabad, India

**Keywords:** gut-liver axis, metabolic associated fatty liver disease, metabolic syndrome, pharmacological targets, precision nutrition, public health, resmetirom, SLC11A1

Metabolic-Associated Fatty Liver Disease (MAFLD) has rapidly become a leading global health challenge of the 21st century, currently affecting around a quarter of the adult population worldwide (Younossi et al., 2025). As a systemic metabolic disorder, MAFLD is strongly associated with obesity and type 2 diabetes mellitus (T2DM), as well as an increased risk of cardiovascular mortality (Zhou et al., 2023). Addressing the MAFLD epidemic is not only a clinical necessity, but also a critical step towards achieving United Nations Sustainable Development Goal 3 (SDG 3), which aims to ensure healthy lives and promote wellbeing at all ages (United Nations, 2015). In this context, the present Research Topic, titled “Pharmacological and Nutritional Approaches to Metabolic-Associated Fatty Liver Disease”, brings together five studies that collectively highlight emerging therapeutic perspectives.

The management of MAFLD remains largely reliant on lifestyle and nutritional modifications as the most broadly applicable intervention strategies. In this context, Yu et al. provide important evidence from a large hospital-based cohort in China, examining the association between sugar-sweetened beverage (SSB) consumption and MAFLD risk among individuals with type 2 diabetes mellitus. In 3,305 patients, weekly SSB intake was analysed using multivariable logistic regression models. Compared with those consuming none or fewer than one SSB per week, individuals consuming 3–4 bottles weekly had significantly higher odds of MAFLD (adjusted OR = 1.72, 95%CI: 1.25–2.58; p = 0.004), with a clear dose–response relationship across higher intake categories (p for trend <0.001). These associations remained robust after adjustment for major demographic and metabolic covariates. Mechanistically, the findings support a role for excessive SSB intake in hepatic *de novo lipogenesis* and metabolic dysregulation, highlighting SSB reduction as a practical, low-cost target for nutritional counselling. Notably, the authors emphasise regional variability in SSB consumption trends across Asia, underscoring the importance of population-specific dietary risk assessment.

The real-world clinical and economic relevance of lifestyle-based interventions is further demonstrated by Polignano et al., who evaluated a multidisciplinary, kinesiology-supervised lifestyle programme implemented in routine care for adults with metabolic dysfunction-associated steatotic liver disease (MASLD) in Italy. This pre–post observational study included 27 adults (≥18 years) with ultrasound-confirmed MASLD who completed a 12-month intervention combining thrice-weekly supervised aerobic and resistance exercise with individualized dietary counselling. Exercise sessions were delivered in a hospital-affiliated gym under continuous professional supervision, while nutritional guidance was provided by a dietitian and tailored to individual metabolic needs. The programme resulted in significant improvements in health-related quality of life (QALY gain: 0.081, 95%CI: 0.001–0.161), reductions in systolic and diastolic blood pressure (−5.6/−3.7 mmHg; p = 0.05 and p = 0.03), and favourable liver-related outcomes, including a shift toward lower ultrasound steatosis grades (p = 0.007) and declines in serum aminotransferases (p < 0.01). Importantly, cost–utility analysis indicated a favourable incremental cost-effectiveness ratio (€17,778 per QALY), well below accepted willingness-to-pay thresholds, supporting supervised lifestyle programmes as a cost-effective and scalable component of MASLD management.

In addition to lifestyle factors, the systemic complexity of MAFLD necessitates a broader understanding of its interactions with endocrine and metabolic pathways. In this context, Xiong et al. conducted a systematic review and meta-analysis examining the association between thyroid hormone profiles and MAFLD. Rather than relying on clinical thyroid diagnoses, abnormal thyroid function was defined based on circulating hormone parameters, including TSH, FT3, FT4, TT3, and TT4. The pooled analysis showed that individuals with MAFLD had significantly higher TSH levels (SMD = 0.02, 95% CI: 0.01–0.03) and markedly lower FT4 levels (SMD = −0.41, 95%CI: −0.42 to −0.40), alongside higher FT3 and TT3 and lower TT4 concentrations. Collectively, these robust associations indicate a higher likelihood of hepatic steatosis in individuals with altered thyroid hormone profiles and support the integration of thyroid function assessment into MAFLD risk stratification beyond conventional metabolic markers.

There is an increasing focus on targeting the gut–liver axis in emerging therapeutic strategies, as it is recognised as a critical modulator of metabolic and inflammatory processes in MAFLD. In this context, Teofilović et al. assessed the therapeutic potential of the probiotic strain *Lactobacillus helveticus* BGRA43 using a high-fat diet–induced obesity model in male C57BL/6J mice. They found that oral administration of this probiotic strain significantly reduced hepatic lipid accumulation and restored gut microbiota diversity. By emphasising the role of functional foods and so-called ‘psychobiotics’ in modulating systemic inflammation and metabolic homeostasis, this study highlights the potential of accessible nutraceutical interventions. Such approaches are particularly relevant for advancing health equity, especially in settings where access to advanced pharmacological therapies remains limited.

A study has identified new diagnostic and therapeutic targets for MAFLD. Wang et al. used advanced bioinformatics and machine learning techniques to determine that SLC11A1 and ERN1 are important regulators of iron metabolism and ferroptosis in MAFLD. Integrated analyses combining transcriptomic profiling, single-cell RNA sequencing and experimental validation showed that dysregulated SLC11A1 expression leads to hepatic iron imbalance, oxidative stress and immune-mediated liver injury. Interestingly, SLC11A1 showed strong diagnostic performance and distinct expression patterns in different cell types within the hepatic immune microenvironment. Furthermore, molecular docking and molecular dynamics simulations provided mechanistic insight into the potential modulation of ferroptosis by emerging pharmacological therapies by revealing stable binding between resmetirom and SLC11A1. Taken together, these results suggest that SLC11A1 could be used as a biomarker and a therapeutic target in precision-based MAFLD management.

In conclusion, the research presented in this Research Topic underscores the need for coordinated action across clinical practice, public health policy, and translational research to advance progress toward Sustainable Development Goal three in the context of MAFLD. The findings indicate that effective MAFLD prevention and management must extend beyond clinical care to include population-level dietary policies, early risk stratification, and equitable access to innovative therapies. By integrating evidence on dietary exposures, endocrine and gut–liver interactions, and molecular targets, this Research Topic provides a robust scientific foundation for evidence-based health policy and health system planning. We sincerely thank all authors and reviewers whose contributions have been instrumental in bridging science, policy, and practice to address the global burden of metabolic liver disease.

## References

[B1] United Nations (2015). Transforming our world: the 2030 agenda for sustainable development. New York: United Nations. Available online at: https://sdgs.un.org/goals/goal3 (Accessed February 13, 2026).

[B2] Younossi Z. M. Kalligeros M. Henry L. (2025). Epidemiology of metabolic dysfunction-associated steatotic liver disease. Clin. Mol. Hepatology 31 (Suppl. l), S32–S50. 10.3350/cmh.2024.0431 39159948 PMC11925440

[B3] Zhou X. D. Targher G. Byrne C. D. Somers V. Kim S. U. Chahal C. A. A. (2023). An international multidisciplinary consensus statement on MAFLD and the risk of CVD. Hepatol. Int. 17 (4), 773–791. 10.1007/s12072-023-10543-8 37204656 PMC10198034

